# Synthesis of 2-amino-3-arylpropan-1-ols and 1-(2,3-diaminopropyl)-1,2,3-triazoles and evaluation of their antimalarial activity

**DOI:** 10.3762/bjoc.7.205

**Published:** 2011-12-30

**Authors:** Matthias D’hooghe, Stéphanie Vandekerckhove, Karen Mollet, Karel Vervisch, Stijn Dekeukeleire, Liesbeth Lehoucq, Carmen Lategan, Peter J Smith, Kelly Chibale, Norbert De Kimpe

**Affiliations:** 1 Department of Sustainable Organic Chemistry and Technology, Faculty of Bioscience Engineering, Ghent University, Coupure Links 653, B-9000 Ghent, Belgium; 2 Medical School, University of Cape Town, K45, OMB, Groote Schuur Hospital, Observatory, 7925, South Africa; 3 Department of Chemistry and Institute of Infectious Disease & Molecular Medicine, University of Cape Town, Rondebosch 7701, South Africa

**Keywords:** aminopropanes, antimalarial activity, aziridines, β-lactams, ring opening

## Abstract

A variety of 2-amino-3-arylpropan-1-ols, *anti*-2-amino-3-aryl-3-methoxypropan-1-ols and *anti*-2-amino-1-arylpropan-1,3-diols were prepared selectively through elaboration of *trans*-4-aryl-3-chloro-β-lactams. In addition, a number of 2-(azidomethyl)aziridines was converted into novel 2-[(1,2,3-triazol-1-yl)methyl]aziridines by Cu(I)-catalyzed azide-alkyne cycloaddition, followed by microwave-assisted, regioselective ring opening by dialkylamine towards 1-(2,3-diaminopropyl)-1,2,3-triazoles. Although most of these compounds exhibited weak antiplasmodial activity, six representatives showed moderate antiplasmodial activity against both a chloroquine-sensitive and a chloroquine-resistant strain of *Plasmodium falciparum* with IC_50_-values of ≤25 μM.

## Introduction

**Figure 1:**
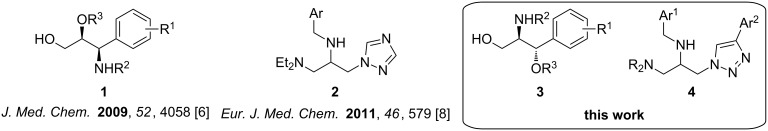
Functionalized aminopropanes as potential antimalarial agents.

Malaria remains a major issue in health control, especially in developing countries. This disease affects 40% of the global population, causing an annual mortality of one million people [1]. Despite recent advances in the development of a vaccine against malaria, chemotherapy remains the most viable alternative towards treatment of the disease [2]. In light of the rapid emergence of multiple drug resistance to clinically established antimalarial drugs, however, there is a compelling need to introduce new chemicals that can overcome this resistance. In 2007, nitrogen-analogues of glycerol, which have a long-standing tradition in medicine as β-blockers, were introduced as a novel class of antimalarials [3]. Prior to this, the well known β-blocker propranolol was shown to inhibit infection of erythrocytes by *P. falciparum*, as well as to reduce the parasitaemia of *P. berghei* infections in vivo [4–5]. In light of the biological potential of these compounds, continuous efforts have been devoted to the preparation of structurally diverse analogues bearing a functionalized propane skeleton [6–8]. In that respect, we have been engaged in the stereoselective synthesis of *syn*-2-alkoxy-3-amino-3-arylpropan-1-ols **1** by reductive ring opening of the corresponding β-lactams, which were shown to be of great importance as novel antiplasmodial agents (Figure 1) [6]. More recently, we reported 1,2,3-triaminopropanes **2** as a new class of antimalarial compounds (Figure 1), prepared through microwave-assisted, regioselective ring opening of the corresponding 2-(aminomethyl)aziridines by diethylamine [8]. Nonetheless, a number of challenges with regard to structure–activity relationship studies of functionalized aminopropanes remain unaddressed, especially concerning the screening of structural analogues of aminopropanes **1** and **2**.

In the present paper, the synthesis of racemic *anti*-2-aminopropan-1-ols **3** is described as a variant of the *syn*-3-aminopropan-1-ols **1** synthesis (regio- and stereoisomerism with respect to the relative position of the amino group NHR^2^ and the oxygen substituent OR^3^, Figure 1), by applying a different synthetic route. Furthermore, a new synthetic approach is disclosed towards racemic aminopropanes **4** bearing a 1,2,3-triazole moiety, as structural analogues of the previously reported 1,2,4-triazoles **2** (Figure 1). Both classes of functionalized aminopropanes **3** and **4** were tested for their antiplasmodial activity.

## Results and Discussion

### Synthesis

**Scheme 1:**
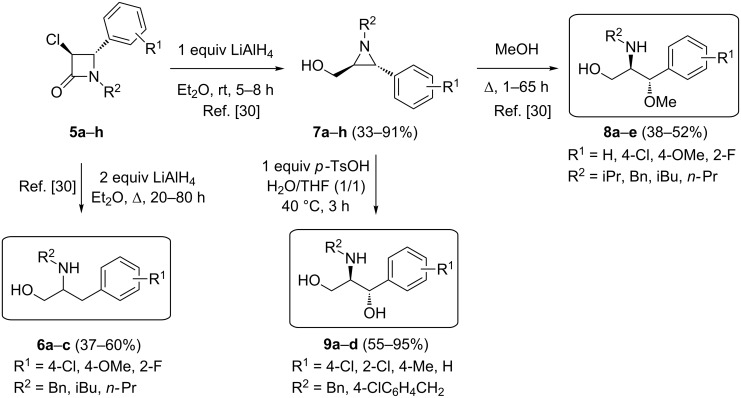
Synthesis of aminopropanols **6**, **8** and **9**.

Within azaheterocyclic chemistry, aziridines [9–17] and β-lactams [18–27] are extraordinary classes of strained compounds with diverse synthetic and biological applications. In previous works, we have elaborated the synthetic potential of 3-chloroazetidin-2-ones with a focus on stereoselectivity, thus providing convenient entries into, e.g., aziridines, azetidines and β-aminoalcohols [28–31]. In continuation of our interest in the use of functionalized β-lactams as synthons for further elaboration, racemic *trans*-4-aryl-3-chloro-β-lactams **5** were prepared by treatment of *N*-(arylmethylidene)alkylamines (synthesized in high yields through condensation of the corresponding benzaldehydes with the appropriate primary amines in CH_2_Cl_2_ in the presence of anhydrous MgSO_4_) with 1.5 equiv of chloroacetyl chloride and 3 equiv of 2,6-lutidine in benzene according to a literature protocol [30]. Subsequently, β-lactams **5** were subjected to LiAlH_4_-mediated reductive ring opening, furnishing either 2-aminopropan-1-ols **6a**–**c**, by using two molar equiv of LiAlH_4_ in Et_2_O under reflux for 20–80 h, or *trans*-2-aryl-3-(hydroxymethyl)aziridines **7a**–**h** by applying milder reactions conditions (i.e., one molar equiv of LiAlH_4_ in Et_2_O at room temperature for 5–8 h) (Scheme 1) [30].

As aziridines are known to be versatile synthetic intermediates for the preparation of a variety of ring-opened and ring-expanded amines, the aziridines **7** were deployed as substrates for the stereoselective synthesis of functionalized aminopropanols. In accordance with a literature approach [30], the nonactivated *trans*-2-aryl-3-(hydroxymethyl)aziridines **7** were regio- and stereoselectively converted into *anti*-2-amino-3-aryl-3-methoxypropan-1-ols **8a**–**e** through heating in methanol under reflux (Scheme 1). Furthermore, in order to provide access to the class of 2-aminopropan-1,3-diols, aziridines **7** were evaluated for the first time as substrates for a water-induced aziridine ring opening in an acidic medium. Thus, treatment of *trans*-2-aryl-3-(hydroxymethyl)aziridines **7** with one equiv of *para*-toluenesulfonic acid in a H_2_O/THF (1/1) solvent system [32] furnished novel *anti*-2-amino-1-arylpropan-1,3-diols **9a**–**d** in good yields after 3 h at 40 °C, again in a regio- and stereospecific way (Scheme 1). The observed regio- and stereoselectivity in aminopropanols **8** and **9** can be rationalized by considering the ring opening of the aziridine moiety at C2 due to benzylic stabilization of the developing carbenium ion in an S_N_2 fashion [30].

**Scheme 2:**
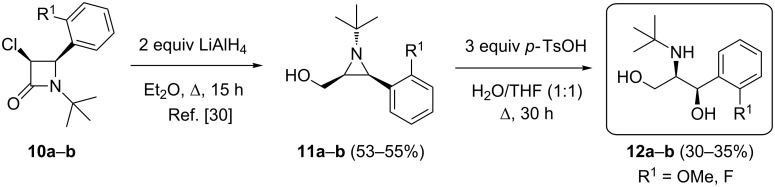
Synthesis of *syn*-aminopropan-1,3-diols **12**.

As the use of imines bearing a *N*-*tert*-butyl group in combination with a substituent in the *ortho*-position of the aromatic ring is known to afford the corresponding *cis*-4-aryl-3-chloroazetidin-2-ones as the major stereoisomers after condensation with chloroketene in benzene [30], racemic *cis*-3-chloro-β-lactams **10a**,**b** were prepared and converted into *cis*-2-aryl-3-(hydroxymethyl)aziridines **11a**,**b** upon treatment with two molar equiv of LiAlH_4_ in Et_2_O under reflux for 15 h (Scheme 2). Next, the aziridines **11**, which have previously been shown to be unreactive towards LiAlH_4_ and methanol and thus unable to undergo ring opening [30], were used as substrates for a water-induced ring opening through initial protonation of the aziridine ring with *p*-TsOH. Although more drastic reaction conditions were required compared to the ring opening of *trans*-2-aryl-3-(hydroxymethyl)aziridines **7** (3 equiv *p*-TsOH, Δ, 30 h instead of 1 equiv *p*-TsOH, 40 °C, 3 h), novel *syn*-aminopropanols **12a**,**b** were obtained in a selective and convenient way (Scheme 2, yields after purification), providing the first example of the ring opening of this type of aziridines. Also in this case, the observed regio- and stereoselectivity in the formation of aminopropanols **12** can be rationalized by considering the ring opening of the aziridine moiety at C2 due to benzylic stabilization of the developing carbenium ion in an S_N_2 fashion [30]. The formation of the other regio- and stereoisomers was excluded based on detailed spectroscopic analysis.

It should be noted that both diastereomeric antipodes of the class of 1-aryl-2-aminopropan-1,3-diols, i.e., *anti*- and *syn*-aminopropanols **9** and **12**, can now be prepared selectively through choice of the appropriate imine for the Staudinger synthesis of the starting β-lactams.

Given the recently disclosed antiplasmodial activities of a number of 2,3-diamino-1-(1,2,4-triazol-1-yl)propanes [8], the second objective of this work was the preparation of new analogues bearing a 1,2,3-triazole moiety instead. A powerful methodology towards the synthesis of functionalized 1,2,3-triazoles involves the Cu(I)-catalyzed azide-alkyne Huisgen cycloaddition (CuAAC) [33], which has gained major interest from the synthetic community due to its high efficiency and selectivity. Eligible substrates to perform “click chemistry” [34] incorporate an azide group and an aziridine ring in their structure, for example in 2-(azidomethyl)aziridines, thus providing a direct access to 2-[(1,2,3-triazol-1-yl)methyl]aziridines through Cu-catalyzed reaction with alkynes [35].

**Scheme 3:**
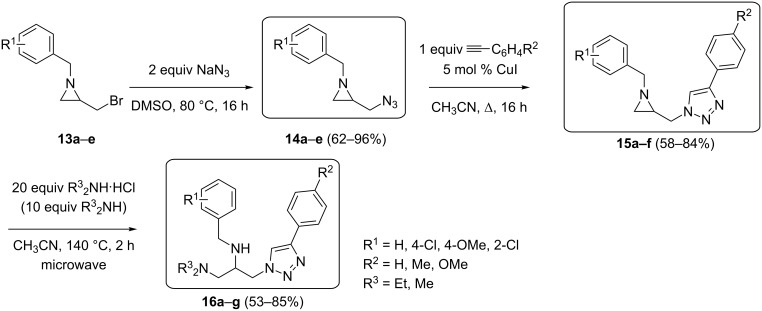
Synthesis of 2-[(1,2,3-triazol-1-yl)methyl]aziridines **15** and their transformation into 1,2,3-triaminopropanes **16**.

In this work, nonactivated *N*-(arylmethyl)aziridines were selected as substrates, as previous research had revealed the importance of *N*-benzyl, *N*-chlorobenzyl and *N*-methoxybenzyl groups in functionalized aminopropanes with regard to their antiplasmodial activity [6,8]. Thus, a number of racemic 2-(azidomethyl)aziridines **14** was prepared by reaction of sodium azide with 2-(bromomethyl)aziridines **13** [36–38], employing the electrophilicity of the latter as a convenient handle for their connection to other moieties. In this way, novel 2-(azidomethyl)aziridines **14a**–**e** were prepared in good yields by treatment of bromides **13** with two equiv of sodium azide in DMSO at 80 °C for 16 h (CAUTION) (Scheme 3). Subsequently, a CuI-catalyzed 1,3-cycloaddition of *N*-(arylmethyl)aziridine azides **14** was evaluated for the first time by utilizing one equiv of an arylacetylene in CH_3_CN under reflux for 16 h, furnishing a direct entry towards new 2-[(1,2,3-triazol-1-yl)methyl]aziridines **15a**–**f** in a highly efficient and selective way (Scheme 3).

The final step comprised ring opening of the aziridine moiety in compounds **15** by diethylamine to afford functionalized aminopropanes as potential antimalarial agents. With the intention to introduce a diethylamino group, a microwave-promoted ring opening of analogous aziridines by diethylamine with the aid of the Et_2_NH·HCl/Et_2_NH system was employed [8]. In this way, protonation of the aziridine ring provides a highly electrophilic aziridinium intermediate, which is prone to undergo nucleophilic ring opening. In order to drive the reaction to completion, and to avoid competition between chloride- and diethylamine-induced ring opening, a large excess of Et_2_NH·HCl (20 equiv) and an additional amount of diethylamine (10 equiv) was used. Thus, heating of the aziridines **15** at 140 °C in CH_3_CN under microwave irradiation resulted in full and selective conversion to the desired new triaminopropanes **16a**–**e** after 2 h (Scheme 3), which were purified by column chromatography (SiO_2_) in order to obtain analytically pure samples. Furthermore, in addition to the use of diethylamine, the introduction of a dimethylamino group was considered in order to compare the contribution of this moiety to the potential antiplasmodial activity with that of a diethylamino group. This objective was achieved by treatment of aziridines **15** with 20 equiv of Me_2_NH·HCl in CH_3_CN at 140 °C for 2 h under microwave irradiation, resulting in dimethylaminopropanes **16f**,**g** in good yields (Scheme 3). This result showed that this microwave-assisted methodology for the ring opening of nonactivated aziridines can be further extended towards the use of other secondary amines. In order to introduce structural diversity within these molecules, different substituent patterns at the aromatic rings (R^1^, R^2^) in aminopropanes **16a**–**g** were realized as well.

In view of the biological potential of aminopropanes in general, compounds **6**, **8**, **9**, **12** and **16** were subsequently screened for their antiplasmodial activity.

In addition, aziridines **14** and **15** were tested against the malaria parasite *Plasmodium falciparum*, too.

### Biological evaluation

At first, compounds **6a**–**c**, **8a**–**e**, **9a**–**d**, **12a**,**b**, **14a**–**e**, **15a**–**f** and **16a**–**g** were screened for in vitro antiplasmodial activity. All samples were tested in triplicate on one occasion against a chloroquine-sensitive (CQS) strain of *P. falciparum* (D10). Those samples showing antiplasmodial activity were then tested in triplicate on one occasion against a chloroquine-resistant (CQR) strain of *P. falciparum* (Dd2) and screened for in vitro cytotoxicity against a Chinese hamster ovary (CHO) cell-line, in triplicate on one occasion. The antiplasmodial and cytotoxicity assays were performed as described previously [8,39–40].

The results from the biological study are summarized in Table 1. Although most of these compounds were shown to possess weak or no antiplasmodial activity, eight of them (i.e., compounds **6a**, **9a**,**b**, **15c**,**d**, **16a**,**b**,**f**) were identified as potentially interesting for further study with IC_50_-values of ≤25 μM. Moreover, these compounds, with the exception of triaminopropanes **16a**,**b**, also proved to be active against a chloroquine-resistant strain of *P. falciparum* (Dd2). In addition, the in vitro cytotoxicity results showed that only compounds **6a** and **16f** have lower selectivity with SI’s of 11 and 16, respectively, whereas the other compounds did not show cytotoxicity at the concentrations tested.

**Table 1:** IC_50_-values of compounds **6**, **8**, **9**, **12**, **14**, **15** and **16** tested for in vitro antiplasmodial activity and cytotoxicity.

Compound	R^1^	R^2^	R^3^	D10: IC_50_ (μM)	Dd2: IC_50_ (μM)	CHO: IC_50_ (μM)	RI^a^	SI^b^

**6a**	4-Cl	Bn	–	12.58	10.88	137.90	0.9	11
**6b**	4-OMe	iBu	–	281.58	ND	ND	ND	ND
**6c**	2-F	*n*-Pr	–	217.34	ND	ND	ND	ND
**8a**	H	iPr	–	369.70	ND	ND	ND	ND
**8b**	H	Bn	–	143.79	ND	ND	ND	ND
**8c**	4-Cl	Bn	–	198.56	ND	ND	ND	ND
**8d**	4-OMe	iBu	–	38.56	25.69	>530	0.7	ND
**8e**	2-F	*n*-Pr	–	38.54	21.92	>530	0.6	ND
**9a**	4-Cl	Bn	–	25.22	8.47	>530	0.3	ND
**9b**	2-Cl	Bn	–	21.18	13.57	>530	0.6	ND
**9c**	4-Me	Bn	–	129.86	ND	ND	ND	ND
**9d**	H	4-ClBn	–	80.68	ND	ND	ND	ND
**12a**	OMe	–	–	98.68	ND	ND	ND	ND
**12b**	F	–	–	60.46	ND	ND	ND	ND
**14a**	H	–	–	230.40	466.81	>530	2	ND
**14b**	4-Cl	–	–	100.82	ND	ND	ND	ND
**14c**	4-OMe	–	–	160.50	ND	ND	ND	ND
**14d**	2-Cl	–	–	93.68	ND	ND	ND	ND
**14e**	3-Cl	–	–	179.86	ND	ND	ND	ND
**15a**	H	H	–	34.44	106.97	>530	3.1	ND
**15b**	4-Cl	H	–	41.32	ND	ND	ND	ND
**15c**	4-OMe	H	–	25.69	55.65	>530	2.2	ND
**15d**	H	Me	–	20.43	20.99	>530	1	ND
**15e**	H	OMe	–	40.54	ND	ND	ND	ND
**15f**	2-Cl	H	–	32.33	25.00	>530	0.8	ND
**16a**	H	H	Et	22.09	231.47	>530	10.5	ND
**16b**	4-Cl	H	Et	25.86	176.40	>530	6.8	ND
**16c**	4-OMe	H	Et	139.61	ND	ND	ND	ND
**16d**	H	Me	Et	32.55	ND	ND	ND	ND
**16e**	H	OMe	Et	171.37	ND	ND	ND	ND
**16f**	2-Cl	H	Me	11.33	13.03	181.83	1.2	16.1
**16g**	H	H	Me	69.58	ND	ND	ND	ND

CQ				19.14 ng/mL (*n* = 6)	75.56 ng/mL (*n* = 5)		3.9	
Emetine						0.27 (*n* = 6)		

^a^RI (Resistance Index) = IC_50_ Dd2/IC_50_ D10; ^b^SI (Selectivity Index) = IC_50_ CHO/IC_50_ D10; ND = not determined; *n* = number of data sets averaged. The more hydrophobic samples were added to the parasites as a suspension, meaning that for these samples the reported IC_50_-value might be an underestimation of the activity.

Although the aziridine moiety was initially only considered as a synthetically useful entity, two 2-[(1,2,3-triazol-1-yl)methyl]aziridines (**15c** and **15d**) were also found to exhibit weak antiplasmodial activity. On the other hand, the aminopropane unit has again proven its value as a template for the preparation of novel antimalarial agents, as a variety of structurally different aminopropanes were demonstrated to exhibit weak to moderate antiplasmodial activity. In particular, antiplasmodial assays against a chloroquine-sensitive strain of *P. falciparum* (D10) showed activity for 2-aminopropan-1-ol **6a**, 2-aminopropan-1,3-diols **9a**,**b** and triaminopropanes **16a**,**b**,**f** with IC_50_-values between 11.3 and 25.9 μM. Moreover, screening against a chloroquine-resistant strain of *P. falciparum* (Dd2) revealed antiplasmodial activity for 2-aminopropan-1-ol **6a**, 2-aminopropan-1,3-diols **9a**,**b** and triaminopropane **16f** with IC_50_-values between 8.5 and 13.6 μM.

From a structure–activity relationship viewpoint, the presence of a chlorinated aromatic ring seems to contribute to the antiplasmodial activity of these functionalized aminopropanes, and the introduction of a dimethylamino group at the expense of a diethylamino moiety might provide better activities in some cases. It is noteworthy that these compounds were synthesized in racemic form, and it is conceivable that enantiomerically pure variants could deliver superior activities. It should also be noted that, in general, the bioactivities reported in this paper are less pronounced as compared to those described in literature precedents on the synthesis and evaluation of functionalized aminopropanes [6,8].

## Conclusion

In summary, a variety of 2-amino-3-arylpropan-1-ols, *anti*-2-amino-3-aryl-3-methoxypropan-1-ols and *anti*-2-amino-1-arylpropan-1,3-diols were prepared selectively through elaboration of *trans*-4-aryl-3-chloro-β-lactams. Furthermore, a number of 2-(azidomethyl)aziridines were converted into novel 2-[(1,2,3-triazol-1-yl)methyl]aziridines by Cu(I)-catalyzed azide-alkyne cycloaddition, followed by microwave-assisted, regioselective ring opening by diethyl- or dimethylamine towards the corresponding 1-(2,3-diaminopropyl)-1,2,3-triazoles. From a synthetic viewpoint, new insights were provided concerning the water-induced ring opening of nonactivated *cis*- and *trans*-2-aryl-3-(hydroxymethyl)aziridines and with respect to the synthesis and use of 1-arylmethyl-2-(azidomethyl)aziridines for azide-alkyne cycloaddition reactions. From a biological viewpoint, most of these compounds exhibited weak antiplasmodial activity, although six representatives showed moderate antiplasmodial activity against both a chloroquine-sensitive and a chloroquine-resistant strain of *P. falciparum* with IC_50_-values of ≤25 μM.

## Experimental

General information regarding NMR, IR, MS and elemental analyses, melting point measurements, and microwave reaction conditions can be found in the literature [8].

### *anti*-2-(*N*-Benzylamino)-1-(4-chlorophenyl)propan-1,3-diol (**9a**)

Recrystallization from hexane/EtOAc (1:25), white crystals, 95%. Mp 192.3 °C; ^1^H NMR (300 MHz, CDCl_3_) δ 2.39 (s, 2H), 3.12–3.18 (m, 1H), 3.54 (dd, *J* = 13.0, 3.6 Hz, 1H), 3.82 (dd, *J* = 13.0, 5.8 Hz, 1H), 4.27 and 4.32 (2 d, *J* = 13.2 Hz, 2 × 1H), 5.24 (d, *J* = 2.2 Hz, 1H), 7.04–7.07, 7.19–7.36, 7.46–7.49 and 7.69–7.71 (4 m, 2H, 3H, 2H, 2H); ^13^C NMR (75 MHz, CDCl_3_) δ 51.0, 59.4, 62.6, 72.0, 127.2, 128.1, 128.7, 128.8, 128.9, 133.4, 137.0, 139.1; IR (cm^−1^) ν_max_: 3437 (OH), 3342 (NH), 2926, 1448, 1162, 1008, 681; MS (70 eV) *m*/*z* (%): 292/4 (M^+^ + 1, 100); HRMS (ESI): [M + H]^+^ calcd for C_16_H_19_ClNO_2_, 292.1104; found, 292.1112.

### *syn*-2-(*N*-*tert*-Butylamino)-1-(2-methoxyphenyl)propan-1,3-diol (**12a**)

*R*_f_ 0.07 (EtOAc), white crystals, 30%. Mp 112.8 °C; ^1^H NMR (300 MHz, CDCl_3_) δ 1.03 (s, 9H), 2.77 (ddd, *J* = 7.1, 3.0, 3.0 Hz, 1H), 3.50–3.51 (m, 2H), 3.86 (s, 3H), 4.80 (d, *J* = 7.1 Hz, 1H), 6.87–6.89, 6.98–7.03, 7.22–7.28 and 7.47–7.50 (4 m, 4 × 1H); ^13^C NMR (75 MHz, CDCl_3_) δ 30.0, 50.8, 55.6, 58.1, 63.7, 68.5, 110.4, 121.3, 127.7, 128.4, 130.5, 156.5; IR (cm^−1^) ν_max_: 3391 (NH), 3313 (OH), 3058, 3004, 2987, 2957, 2927, 2873, 2838, 1492, 1467, 1438, 1368, 1242, 1066, 1050, 1027, 755; MS (70 eV) *m*/*z* (%): 254 (M^+^ + 1, 100); HRMS (ESI): [M + H]^+^calcd for C_14_H_24_NO_3_, 254.1756; found, 254.1766.

### 2-Azidomethyl-1-(phenylmethyl)aziridine (**14a**)

*R*_f_ 0.20 (hexane/EtOAc 4:1), yellow oil, 70%. ^1^H NMR (300 MHz, CDCl_3_) δ 1.51 (d, *J* = 6.1 Hz, 1H), 1.79 (d, *J* = 3.3 Hz, 1H), 1.79–1.86 (m, 1H), 3.18 and 3.27 (2 dd, *J* = 12.9, 6.6, 4.4 Hz, 2H) 3.38 and 3.58 (2 d, *J* = 13.2 Hz, 2H), 7.24–7.36 (m, 5H); ^13^C NMR (75 MHz, CDCl_3_) δ 32.2, 37.9, 53.8, 64.3, 127.4, 128.3, 128.6, 138.8; IR (cm^−1^) ν_max_: 2088 (N_3_), 1453, 1357, 1322, 1255, 1159, 1062, 1028, 907, 732, 697; MS (70 eV) *m*/*z* (%): 189 (M^+^ + 1, 100); HRMS (ESI): [M + H]^+^ calcd for C_10_H_13_N_4_, 189.1140; found, 189.1141.

### 1-Phenylmethyl-2-[(4-phenyl-1,2,3-triazol-1-yl)methyl]aziridine (**15a**)

*R*_f_ 0.23 (CHCl_3_/MeOH 98:2), viscous light-brown oil, 61%. ^1^H NMR (300 MHz, CDCl_3_) δ 1.65 (d, *J* = 6.6 Hz, 1H), 1.88 (d, *J* = 3.3 Hz, 1H), 2.01–2.08 (m, 1H), 3.09 and 3.67 (2 d, *J* = 12.9 Hz, 2H), 3.93 and 4.70 (2 dd, *J* = 14.3, 8.2, 3.3 Hz, 2H), 7.06–7.43 (m, 8H), 7.49 (s, 1H), 7.66–7.69 (m, 2H); ^13^C NMR (75 MHz, CDCl_3_) δ 32.4, 38.3, 53.5, 64.5, 119.9, 125.9, 127.7, 128.1, 128.4, 128.6, 128.7, 130.8, 138.2, 147.9; IR (cm^−1^) ν_max_: 2919, 1453, 1358, 1225, 1075, 1046, 1027, 763, 731, 694; MS (70 eV) *m*/*z* (%): 291 (M^+^ + 1, 100); HRMS (ESI): [M + H]^+^ calcd for C_18_H_19_N_4_, 291.1610; found, 291.1613.

### 3-Diethylamino-2-(phenylmethyl)amino-1-(4-phenyl-1,2,3-triazol-1-yl)propane (**16a**)

*R*_f_ 0.19 (CHCl_3_/MeOH 97:3), light-brown oil, 56%. ^1^H NMR (300 MHz, CDCl_3_) δ 0.92 (t, *J* = 7.2 Hz, 6H), 2.26–2.52 (m, 6H), 3.05–3.13 (m, 1H), 3.74 and 3.81 (2 d, *J* = 13.5 Hz, 2H), 4.36 and 4.44 (2 dd, *J* = 14.2, 4.9, 4.7 Hz, 2H), 7.21–7.44 and 7.82–7.84 (2 m, 10H), 7.86 (s, 1H); ^13^C NMR (75 MHz, CDCl_3_) δ 11.8, 47.1, 52.0, 52.3, 55.0, 55.4, 121.2, 125.8, 127.2, 128.1, 128.2, 128.6, 128.9, 130.9, 140.2, 147.5; IR (cm^−1^) ν_max_: 2967, 1462, 1454, 1073, 762, 734, 694; MS (70 eV) *m*/*z* (%): 364 (M^+^ + 1, 100); HRMS (ESI): [M + H]^+^ calcd for C_22_H_30_N_5_, 364.2501; found, 364.2503.

## Supporting Information

File 1 Experimental procedures and characterization data.
