# Supplementary material for: Synthesis of 2-amino-3-arylpropan-1-ols and 1-(2,3-diaminopropyl)-1,2,3-triazoles and evaluation of their antimalarial activity
Source: Beilstein J Org Chem. 2011 Dec 30;7:1745–52. doi: 10.3762/bjoc.7.205 (PMC3252880; doi:10.3762/bjoc.7.205)
Supplement: File 1 — Experimental procedures and characterization data. [file Beilstein_J_Org_Chem-07-1745-s001.pdf]

Supporting Information

for

Synthesis of 2-amino-3-arylpropan-1-ols and 1-(2,3-diaminopropyl)-1,2,3-triazoles and evaluation of their antimalarial activity

Matthias D'hooghe[1*], Stéphanie Vandekerckhove[1], Karen Mollet[1,§], Karel Vervisch[1], Stijn Dekeukeleire[1,§], Liesbeth Lehoucq[1], Carmen Lategan[2], Peter J. Smith[2], Kelly Chibale[3] and Norbert De Kimpe[1*]

Address: [1]Department of Sustainable Organic Chemistry and Technology, Faculty of Bioscience Engineering, Ghent University, Coupure Links 653, B-9000 Ghent, Belgium; [2]Medical School, University of Cape Town, K45, OMB, Groote Schuur Hospital, Observatory, 7925, South Africa and [3]Department of Chemistry and Institute of Infectious Disease & Molecular Medicine, University of Cape Town, Rondebosch 7701, South Africa

Email: Matthias D'hooghe[*] - matthias.dhooghe@UGent.be,
Norbert De Kimpe - norbert.dekimpe@UGent.be

* Corresponding author

§ Aspirant of the "Research Foundation – Flanders" (FWO-Vlaanderen)

Experimental procedures and characterization data

Synthesis of *trans*-4-aryl-3-chloroazetidin-2-ones 5

trans-4-Aryl-3-chloroazetidin-2-ones **5** were synthesized according to a literature procedure [1].

***trans*-1-Benzyl-3-chloro-4-(2-chlorophenyl)azetidin-2-one (5a)**

R_f = 0.35 (hexane/EtOAc 3/1), brown oil, 74%. ^{1}H NMR (300 MHz, $CDCl_3$): δ 3.93 (1H, d, J = 14.9 Hz), 4.58 and 4.89 (2 × 1H, 2 × d, J = 1.4 Hz), 4.91 (1H, d, J = 14.9 Hz), 7.14–7.43 (9H, m). ^{13}C NMR (75 MHz, ref = $CDCl_3$): δ 45.7, 62.0, 62.8, 127.2, 127.6, 128.3, 128.6, 129.1, 130.3, 130.5, 132.8, 133.8, 134.2, 163.9. IR (cm^{-1}): $\nu_{C=O}$ = 1768, ν_{max} = 2920, 1390,

1037, 753, 698. MS (70 eV) *m/z* (%): 306/8/10 (M^+ + 1, 100). HRMS (ESI) calcd for $C_{16}H_{14}Cl_2NO$ 306.0452 $[M + H]^+$, found 306.0444.

***trans*-1-Benzyl-3-chloro-4-(4-methylphenyl)azetidin-2-one (5b)**

R_f = 0.38 (hexane/EtOAc 3/1), brown oil, 65%. ^1H NMR (300 MHz, $CDCl_3$): δ 2.38 (3H, s), 3.78 (1H, d, *J* = 15.1 Hz), 4.35 and 4.53 (2 × 1H, 2 × d, *J* = 1.4 Hz), 4.86 (1H, d, *J* = 15.1 Hz), 7.11–7.35 (9H, m). ^{13}C NMR (75 MHz, ref = $CDCl_3$): δ 21.4, 44.9, 63.4, 65.2, 126.7, 128.2, 128.6, 129.0, 130.1, 131.7, 134.5, 139.6, 163.8. IR (cm^{-1}): $\nu_{C=O}$ = 1765, ν_{max} = 2921, 1392, 797, 698. MS (70 eV) *m/z* (%): 286/8 (M^+ + 1, 100). HRMS (ESI) calcd for $C_{17}H_{17}ClNO$ 286.0999 $[M + H]^+$, found 286.0998 .

***trans*-3-Chloro-1-(4-chlorobenzyl)-4-phenylazetidin-2-one (5c)**

R_f = 0.36 (hexane/EtOAc 3/1), orange crystals, 62%. ^1H NMR (300 MHz, $CDCl_3$): δ 3.82 (1H, d, *J* = 14.9 Hz), 4.37 and 4.56 (2 × 1H, 2 × d, *J* = 1,6 Hz), 4.80 (1H, d, *J* = 14.9 Hz), 7.05–7.08, 7.21–7.29, 7.33–7.42 (2H, 4H, 3H, 3 × m). ^{13}C NMR (75 MHz, ref = $CDCl_3$): δ 44.4, 63.4, 65.5, 126.8, 129.2, 129.5, 129.7, 130.0, 133.0, 134.2, 134.6, 163.7. IR (cm^{-1}): $\nu_{C=O}$ = 1776, ν_{max} = 2921, 1491, 1398, 795, 702. MS (70 eV) *m/z* (%): 306/8/10 (M^+ + 1, 100). Anal. calcd. for $C_{16}H_{13}Cl_2NO$: C 62.76, H 4.28, N 4.57; found: C 62.52, H 4.46, N 4.44. Mp = 104.1 °C.

<u>Synthesis of *trans*-2-aryl-3-(hydroxymethyl)aziridines 7</u>

trans-2-Aryl-3-(hydroxymethyl)aziridines **7** were synthesized according to a literature procedure [1]. Due to hindered nitrogen inversion, two invertomers can be observed upon NMR analysis. The terms “major invertomer” and “minor invertomer” are used in this section to distinguish between these isomers.

***trans*-1-Benzyl-3-(2-chlorophenyl)-2-(hydroxymethyl)aziridine (7f)**

Recrystallization from hexane-EtOAc (2/25), light-yellow crystals, 44%. HRMS (ESI) calcd for $C_{16}H_{17}ClNO$ 274.0999 $[M + H]^+$, found 274.0998. Mp = 86.2 °C. Ratio major/minor: 61/39.

IR (cm^{-1}): ν_{OH} = 3183, ν_{max} = 2985, 2937, 2844, 1440, 1054, 742, 694. MS (70 eV) *m/z* (%): 274/6 (M^+ + 1, 100).

Major invertomer. ^1H NMR (300 MHz, $CDCl_3$): δ 2.42–2.46 (1H, m), 2.80 (1H, d, *J* = 13.2 Hz), 3.44 (1H, d, *J* = 3.3 Hz), 3.60 (1H, d × d, *J* = 11.5, 5.0 Hz), 3.74 (1H, d, *J* = 13.2 Hz), 3.87–3.92 (1H, m), 7.13–7.47 (9H, m). ^{13}C NMR (75 MHz, ref = $CDCl_3$): 42.9, 44.7, 56.1, 62.5, 126.5, 128.0, 128.3, 128.6, 129.6, 129.9, 131.4, 132.2, 137.3, 139.1.

Minor invertomer. ^{1}H NMR (300 MHz, $CDCl_3$): δ 2.32–2.37 (1H, m), 2.92 (1H, d, *J* = 2.8 Hz), 3.90–4.14 (4H, m), 7.13–7.47 (9H, m). ^{13}C NMR (75 MHz, ref = $CDCl_3$): 43.2, 47.6, 55.8, 59.1, 127.1, 127.3, 127.8, 128.1, 128.6, 128.9, 133.6, 137.2, 139.5.

***trans*-1-Benzyl-2-hydroxymethyl-3-(4-methylphenyl)aziridine (7g)**

Recrystallization from hexane-EtOAc (1/30), white crystals, 33%. Mp = 96.9 °C. Anal. calcd. for $C_{17}H_{19}NO$: C 80.60, H 7.56, N 5.53; found: C 80.46, H 7.50, N 5.45. Ratio major/minor: 93/7.

IR (cm^{-1}): ν_{OH} = 3064, 3026, ν_{max} = 2921, 2829, 1452, 1070, 1045, 696. MS (70 eV) *m*/*z* (%): 254 (M^+ + 1, 100).

Major invertomer. ^{1}H NMR (300 MHz, $CDCl_3$): 2.04 (1H, s(br)), 2.36 (3H, s), 2.44–2.49 (1H, m), 3.11 (1H, d, *J* = 13.8 Hz), 3.29 (1H, d, *J* = 3.9 Hz), 3.45 (1H, d, *J* = 13.8 Hz), 3.54 (1H, d × d, *J* = 11.6, 5.5 Hz), 3.85–3.88 (1H, m), 7.09–7.42 (9H, m). ^{13}C NMR (75 MHz, ref = $CDCl_3$): 21.3, 43.8, 44.0, 55.4, 62.5, 127.1, 128.2, 128.5, 129.0, 130.3, 136.7, 137.9, 139.6.

Minor invertomer. ^{1}H NMR (300 MHz, $CDCl_3$): 2.32 (3H, s), 2.52–2.57 (2H, m), 3.85–4.03 (4H, m), 7.09–7.42 (9H, m). ^{13}C NMR (75 MHz, ref = $CDCl_3$): 21.3, 45.3, 48.1, 55.7, 59.2, 126.2, 127.7, 130.2.

***trans*-1-(4-Chlorobenzyl)-2-hydroxymethyl-3-phenylaziridine (7h)**

R_f = 0.21 (Et_2O/hexane 6/1), unstable upon attempted chromatographic purification, orange oil, 91%. Ratio major/minor: 80/20.

IR (cm^{-1}): ν_{OH} = 3314, ν_{max} = 2917, 1491, 1088, 1014, 698. MS (70 eV) *m*/*z* (%): 274/6 (M^+ + 1, 100).

Major invertomer. ^{1}H NMR (300 MHz, $CDCl_3$): 2.45–2.51 (1H, m), 3.19 (1H, d, *J* = 13.8 Hz), 3.31 (1H, d, *J* = 3.3 Hz), 3.36 (1H, d, *J* = 13.8 Hz), 3.58–3.65 (1H, m), 3.88–3.95 (1H, m), 7.07–7.44 (9H, m). ^{13}C NMR (75 MHz, $CDCl_3$): 43.8, 44.1, 54.8, 62.4, 128.2, 128.4, 128.5, 129.5, 130.4, 132.8, 133.0, 137.9.

Minor invertomer. ^{1}H NMR (300 MHz, $CDCl_3$): 2.45–2.51 (1H, m), 2.56 (1H, d, *J* = 2.8 Hz), 3.75–4.08 (4H, m), 7.07–7.44 (9H, m). ^{13}C NMR (75 MHz, $CDCl_3$): 45.3, 48.3, 59.2, 64.6, 127.7, 128.6, 128.8, 129.0, 129.7, 133.0, 137.5, 138.9.

Synthesis of *anti*-2-amino-1-arylpropan-1,3-diols 9

General procedure. To a solution of *trans*-2-aryl-3-(hydroxymethyl)aziridine **7** (2.45 mmol) in THF/water (1/1) (20 mL) was added *p*-TsOH (2.45 mmol) in a single portion. Subsequently, the resulting solution was heated at 40 °C for 3 h. The reaction mixture was allowed to cool to

room temperature and was then neutralized with a saturated sodium bicarbonate solution. The reaction mixture was extracted with CH_2Cl_2 (3 × 10 mL), the organic layer was dried ($MgSO_4$), and the solvent was removed under reduced pressure affording *anti*-2-amino-1-arylpropan-1,3-diol **9**, which was purified by means of column chromatography on silica gel.

***anti*-2-(*N*-Benzylamino)-1-(2-chlorophenyl)propan-1,3-diol (9b)**

R_f = 0.27 ($CHCl_3$/MeOH 9/1), yellow oil, 70%. ^{1}H NMR (300 MHz, $CDCl_3$): 3.09–3.10 (1H, m), 3.37 (1H, d × d, *J* = 12.1, 3.3 Hz), 3.60 (1H, d × d, *J* = 12.1, 6.0 Hz), 3.97 and 4.07 (2 × 1H, 2 × d, *J* = 13.5 Hz), 5.33 (1H, s(br)), 7.07–7.33, 7.48–7.51 and 7.67–7.70 (7H, 1H, 1H, 3 × m). ^{13}C NMR (75 MHz, ref = $CDCl_3$): 50.2, 57.8, 59.6, 68.7, 127.3, 128.1, 128.7, 129.2, 129.3, 129.5, 130.1, 131.1, 131.7, 136.7. IR (cm^{-1}): ν_{OH} = 3337, ν_{max} = 2859, 1440, 1032, 729, 698. MS (70 eV) *m*/*z* (%): 292/4 (M^+ + 1, 100). HRMS (ESI) calcd for $C_{16}H_{19}ClNO_2$ 292.1104 $[M + H]^+$, found 292.1102.

***anti*-2-(*N*-Benzylamino)-1-(4-methylphenyl)propan-1,3-diol (9c)**

R_f = 0.18 (hexane/EtOAc 1/1), orange oil, 87%. ^{1}H NMR (300 MHz, $CDCl_3$): δ 2.29 (3H, s), 2.87–2.91 (1H, m), 3.48 (1H, d × d, *J* = 11.9, 3.0 Hz), 3.67 (1H, d × d, *J* = 11.9, 5.0 Hz), 3.91–4.07 (2H, m), 4.98 (1H, d, *J* = 3.9 Hz), 7.04–7.26 and 7.64–7.66 (4H, 5H, 2 × m). ^{13}C NMR (75 MHz, ref = $CDCl_3$): δ 21.2, 50.6, 58.8, 62.5, 72.2, 125.9, 128.8, 129.1, 129.2, 129.2, 136.1, 137.2, 137.8. IR (cm^{-1}): ν_{OH} = 3328, ν_{max} = 2922, 1661, 1453, 1177, 1010, 698. MS (70 eV) *m*/*z* (%): 272 (M^+ + 1, 100). HRMS (ESI) calcd for $C_{17}H_{22}NO_2$ 272.1651 $[M + H]^+$, found 272.1644.

***anti*-2-[*N*-(4-Chlorobenzyl)amino]-1-phenylpropan-1,3-diol (9d)**

Recrystallization from hexane-EtOAc (1/25), light-yellow crystals, 55%. ^{1}H NMR: (300 MHz, CD_3OD): δ 3.20–3.22 (1H, m), 3.42 (1H, d × d, *J* = 12.0, 3.3 Hz), 3.70 (1H, d × d, *J* = 12.0, 8.5 Hz), 4.29 and 4.34 (2 × 1H, 2 × d, *J* = 13.5 Hz), 5.12 (1H, d, *J* = 3.3 Hz), 7.11–7.14, 7.18–7.31, 7.37–7.40, 7.45–7.48 and 7.58–7.61 (9H, 5 × m). ^{13}C NMR (75 MHz, ref = CD_3OD): δ 48.4, 57.3, 63.3, 69.3, 125.7, 128.4, 128.5, 129.1, 131.6, 135.4, 139.8, 140.4. IR (cm^{-1}): ν_{OH} = 3364, 3258, ν_{max} = 2938, 1415, 1040, 705. MS (70 eV) *m*/*z* (%) 292/4 (M^+ + 1, 100). HRMS (ESI) calcd for $C_{16}H_{19}ClNO_2$ 292.1104 $[M + H]^+$, found 292.1093. Mp = 199.4 °C.

Synthesis of *syn*-2-amino-1-arylpropan-1,3-diols 12

General procedure. To a solution of *cis*-2-aryl-3-(hydroxymethyl)aziridine (**11**) (2.45 mmol) in THF/water (1/1) (30 mL) was added *p*-TsOH (7.35 mmol) in a single portion. Subsequently, the resulting solution was heated under reflux for 30 h. The reaction mixture was allowed to cool to room temperature and was then neutralized with a saturated sodium bicarbonate solution. The mixture was extracted with CH_2Cl_2 (3 × 20 mL), the organic layer was dried ($MgSO_4$), and the solvent was removed under reduced pressure affording *syn*-2-amino-1-arylpropan-1,3-diol **12**, which was purified by means of column chromatography on silica gel.

***syn*-2-(*N*-*tert*-Butylamino)-1-(2-fluorophenyl)propan-1,3-diol (12b)**

R_f = 0.09 (EtOAc), white crystals, 35%. ^{1}H NMR (300 MHz, $CDCl_3$): δ 0.99 (9H, s), 2.85 (1H, d × d × d, *J* = 6.1, 3.0, 3.0 Hz), 3.11 (3H, s (br)), 3.63–3.64 (2H, m), 4.88 (1H, d, *J* = 6.1 Hz), 6.98–7.05, 7.13–7.18, 7.21–7.28 and 7.49–7.55 (4 × 1H, 4 × m). ^{19}F NMR (282 MHz, $CDCl_3$): δ (−118.93)–(−118.85) (m). ^{13}C NMR (75 MHz, $CDCl_3$): δ 29.7, 50.8, 57.5, 63.5, 67.4, 115.1 (d, *J* = 22.0 Hz), 124.2 (d, *J* = 3.5 Hz), 128.3 (d, *J* = 4.6 Hz), 128.8 (d, *J* = 8.1 Hz), 129.9 (d, *J* = 12.7 Hz), 160.2 (d, *J* = 244.6 Hz). IR (cm^{-1}): $\nu_{NH,OH}$ = 3314, ν_{max} = 2965, 1484, 1456, 1226, 1214, 1074, 1040, 809, 750. MS (70 eV) *m*/*z* (%) 242 (M^+ + 1, 100). HRMS (ESI) calcd for $C_{13}H_{21}FNO_2$ 242.1556 $[M + H]^+$, found 242.1555. Mp = 95.7 °C.

Synthesis of 2-(azidomethyl)aziridines 14

General procedure. To a solution of 1-arylmethyl-2-(bromomethyl)aziridine (**13**) [2-4] (4.4 mmol) in dimethylsulfoxide (40 mL) was added sodium azide (17.6 mmol) in small portions at room temperature (CAUTION; behind a safety shield), and the resulting mixture was stirred for 16 h at 80 °C. Afterwards, the reaction mixture was poured into water (40 mL) and extracted with Et_2O (3 × 30 mL). The combined organic fractions were washed with brine (3 × 30 mL). Drying ($MgSO_4$), filtration of the drying agent and removal of the solvent in vacuo afforded 1-arylmethyl-2-(azidomethyl)aziridine (**14**), which was purified by means of column chromatography on silica gel.

2-Azidomethyl-1-[(4-chlorophenyl)methyl]aziridine (14b)

R_f = 0.18 (hexane/EtOAc 4/1), yellow oil, 62%. ^{1}H NMR (300 MHz, $CDCl_3$): δ 1.50 (1H, d, *J* = 6.1 Hz), 1.79 (1H, d, *J* = 3.3 Hz), 1.80–1.85 (1H, m), 3.16 and 3.30 (2H, 2 × d × d, *J* = 13.3, 6.3, 5.0 Hz), 3.35 and 3.54 (2H, 2 × d, *J* = 13.5 Hz), 7.26–7.33 (4H, m). ^{13}C NMR (75 MHz, $CDCl_3$): δ 32.2, 38.0, 53.7, 63.5, 128.6, 129.5, 133.0, 137.3. IR (cm^{-1}): ν_{azide} =

2091, ν_{max} = 1491, 1258, 1087, 806. MS (70 eV) *m/z* (%): 223/5 (M^+ + 1, 100). HRMS (ESI) calcd for $C_{10}H_{12}ClN_4$ 223.0750 $[M + H]^+$, found 223.0750.

2-Azidomethyl-1-[(4-methoxyphenyl)methyl]aziridine (14c)

R_f = 0.2 (hexane/EtOAc 4/1), yellow oil, 68%. ^{1}H NMR (300 MHz, $CDCl_3$): δ 1.46 (1H, d, *J* = 6.6 Hz), 1.71 (1H, d, *J* = 3.3 Hz), 1.75–1.81 (1H, m), 3.11 and 3.22 (2H, 2 × d × d, *J* = 13.2, 6.9, 5.2 Hz), 3.28 and 3.48 (2H, 2 × d, *J* = 12.9 Hz), 3.76 (3H, s), 6.83–6.88 (2H, m), 7.22–7.26 (2H, m). ^{13}C NMR (75 MHz, $CDCl_3$): δ 31.9, 37.7, 53.8, 55.2, 63.6, 113.9, 129.5, 130.8, 158.9. IR (cm^{-1}): ν_{azide} = 2091, ν_{max} = 1512, 1242, 1034. MS (70 eV) *m/z* (%): 219 (M^+ + 1, 100). HRMS (ESI) calcd for $C_{11}H_{15}N_4O$ 219.1246 $[M + H]^+$, found 219.1254.

2-Azidomethyl-1-[(2-chlorophenyl)methyl]aziridine (14d)

R_f = 0.21 (hexane/EtOAc 5/1), yellow oil, 94%. ^{1}H NMR (300 MHz, $CDCl_3$): δ 1.56 (1H, d, *J* = 6.1 Hz), 1.84 (1H, d, *J* = 3.3 Hz), 1.85–1.93 (1H, m), 3.20 and 3.33 (2H, 2 × d × d, *J* = 13.2, 7.2, 4.4 Hz), 3.49 and 3.70 (2H, 2 × d, *J* = 14.6 Hz), 7.17–7.61 (4H, m). ^{13}C NMR (75 MHz, $CDCl_3$): δ 32.3, 38.1, 53.8, 60.9, 127.0, 128.4, 129.3, 129.6, 133.2, 136.4. IR (cm^{-1}): ν_{azide} = 2091, ν_{max} = 1443, 1260, 1241, 1036, 749. MS (70 eV) *m/z* (%): 223 (M^+ + 1, 100). HRMS (ESI) calcd for $C_{10}H_{12}ClN_4$ 223.0750 $[M + H]^+$, found 223.0744.

2-Azidomethyl-1-[(3-chlorophenyl)methyl]aziridine (14e)

R_f = 0.2 (hexane/EtOAc 5/1), yellow oil, 96%. ^{1}H NMR (300 MHz, $CDCl_3$): δ 1.49 (1H, d, *J* = 6.6 Hz), 1.79 (1H, d, *J* = 3.3 Hz), 1.78–1.85 (1H, m), 3.15 and 3.29 (2H, 2 × d × d, *J* = 13.1, 6.9, 5.8 Hz), 3.30 and 3.56 (2H, 2 × d, *J* = 13.8 Hz), 7.19–7.31 (4H, m). ^{13}C NMR (75 MHz, $CDCl_3$): δ 32.3, 38.0, 53.7, 63.6, 126.3, 127.5, 128.2, 129.8, 134.3, 140.8. IR (cm^{-1}): ν_{azide} = 2089, ν_{max} = 1256, 864, 776, 682. MS (70 eV) *m/z* (%): 223/5 (M^+ + 1, 100). HRMS (ESI) calcd for $C_{10}H_{12}ClN_4$ 223.0750 $[M + H]^+$, found 223.0757.

Synthesis of 2-[(1,2,3-triazol-1-yl)methyl]aziridines 15

General procedure. To a solution of 1-arylmethyl-2-(azidomethyl)aziridine (**14**) (5.3 mmol) in acetonitrile (50 mL) was added the arylacetylene (5.3 mmol) and copper(I) iodide (0.26 mmol) at room temperature. The resulting mixture was heated under reflux for 16 h, after which the reaction mixture was poured into water (50 mL) and extracted with dichloromethane (3 × 50 mL). Drying ($MgSO_4$), filtration of the drying agent and removal of the solvent in vacuo afforded 1-arylmethyl-2-[(1,2,3-triazol-1-yl)methyl]aziridine (**15**), which was purified by means of column chromatography on silica gel.

1-(4-Chlorophenyl)methyl-2-[(4-phenyl-1,2,3-triazol-1-yl)methyl]aziridine (15b)

R_f = 0.21 ($CHCl_3$/MeOH 98/2), light-brown crystals, 84%. ^{1}H NMR (300 MHz, $CDCl_3$): δ 1.65 (1H, d, J = 6.6 Hz), 1.90 (1H, d, J = 3.3 Hz), 2.00–2.10 (1H, m), 3.08 and 3.63 (2H, 2 × d, J = 13.2 Hz), 3.94 and 4.72 (2H, 2 × d × d, J = 14.3, 8.2, 3.3 Hz), 7.06–7.43 (7H, m), 7.52 (1H, s), 7.68–7.70 (2H, m). ^{13}C NMR (75 MHz, $CDCl_3$): δ 32.3, 38.5, 53.4, 63.7, 119.8, 125.8, 128.1, 128.8, 128.9, 129.7, 130.6, 133.6, 136.8, 147.9. IR (cm^{-1}): ν_{max} = 2980, 1487, 1079, 1045, 1014, 857, 808, 768, 749, 708, 700. MS (70 eV) m/z (%): 325/7 (M^+ + 1, 100). HRMS (ESI) calcd for $C_{18}H_{18}ClN_4$ 325.1220 $[M + H]^+$, found 325.1226. Mp = 116.5 °C.

1-(4-Methoxyphenyl)methyl-2-[(4-phenyl-1,2,3-triazol-1-yl)methyl]aziridine (15c)

R_f = 0.2 ($CHCl_3$/MeOH 98/2), light-yellow crystals, 30%. ^{1}H NMR (300 MHz, $CDCl_3$): δ 1.68 (1H, d, J = 6.6 Hz), 1.90 (1H, d, J = 3.3 Hz), 2.01–2.08 (1H, m), 2.86 and 3.79 (2H, 2 × d, J = 12.1 Hz), 3.50 (3H, s), 3.82 and 4.79 (2H, 2 × d × d, J_{gem} = 14.3 Hz, J_{vic} = 8.8 en 3.3 Hz), 6.70–6.74 (2H, m), 7.10–7.13 (2H, m), 7.26–7.44 (3H, m), 7.39 (1H, s), 7.65–7.77 (2H, m). ^{13}C NMR (75 MHz, $CDCl_3$): δ 32.1, 38.3, 53.6, 55.0, 64.0, 114.0, 119.8, 125.7, 128.0, 128.8, 129.8, 130.3, 130.7, 147.8, 159.1. IR (cm^{-1}): ν_{max} = 2922, 1510, 1243, 1227, 1037, 816, 767, 749, 700. MS (70 eV) m/z (%): 321 (M^+ + 1, 100). HRMS (ESI) calcd for $C_{19}H_{21}N_4O$ 321.1715 $[M + H]^+$, found 321.1728. Mp = 91.7 °C.

1-Phenylmethyl-2-{[4-(4-methylphenyl)-1,2,3-triazol-1-yl]methyl}aziridine (15d)

R_f = 0.35 ($CHCl_3$/MeOH 96/4), light-brown oil, 71%. ^{1}H NMR (300 MHz, $CDCl_3$): δ 1.66 (1H, d, J = 6.6 Hz), 1.89 (1H, d, J = 3.3 Hz), 2.02–2.10 (1H, m), 2.38 (3H, s), 3.11 and 3.67 (2H, 2 × d, J = 12.7 Hz), 3.92 and 4.70 (2H, 2 × d × d, J = 14.3, 8.3, 3.8 Hz), 7.09–7.26 (7H, m), 7.46 (1H, s), 7.56–7.58 (2H, m). ^{13}C NMR (75 MHz, $CDCl_3$): δ 21.4, 32.4, 38.3, 53.4, 64.5, 119.6, 125.8, 127.7, 128.4, 128.7, 129.4, 129.6, 137.9 138.2, 147.9. IR (cm^{-1}): ν_{max} = 2922, 822, 799, 751, 730, 698. MS (70 eV) m/z (%): 305 (M^+ + 1, 100). HRMS (ESI) calcd for $C_{19}H_{21}N_4$ 305.1766 $[M + H]^+$, found 305.1756.

1-Phenylmethyl-2-{[4-(4-methoxyphenyl)-1,2,3-triazol-1-yl]methyl}aziridine (15e)

R_f = 0.18 (EtOAc), white crystals, 56%. ^{1}H NMR (300 MHz, $CDCl_3$): δ 1.68 (1H, d, J = 6.1 Hz), 1.91 (1H, d, J = 3.3 Hz), 2.04–2.11 (1H, m), 3.12 and 3.70 (2H, 2 × d, J = 12.7 Hz), 3.86 (3H, s), 3.93 and 4.73 (2H, 2 × d × d, J = 14.3, 8.3, 3.9 Hz), 6.92–6.97 (2H, m), 7.08–7.26 (5H, m), 7.40 (1H, s), 7.58–7.63 (2H, m). ^{13}C NMR (75 MHz, $CDCl_3$): δ 32.4, 38.4, 53.5, 55.4, 64.6, 114.1, 119.1, 123.6, 127.2, 127.8, 128.5, 128.7, 138.2, 147.8, 159.6. IR (cm^{-1}): ν_{max} = 2954, 1247, 1030, 821, 724, 694. MS (70 eV) m/z (%): 321 (M^+ + 1, 100). HRMS (ESI) calcd for $C_{19}H_{21}N_4O$ 321.1715 $[M + H]^+$, found 321.1727. Mp = 91.3 °C.

1-(2-Chlorophenyl)methyl-2-[(4-phenyl-1,2,3-triazol-1-yl)methyl]aziridine (15f)

R_f = 0,21 (hexane/EtOAc 9/1), light-yellow crystals, 68%. ^{1}H NMR (300 MHz, $CDCl_3$): δ 1.72 (1H, d, *J* = 6.6 Hz), 1.97 (1H, d, *J* = 3.3 Hz), 2.14–2.21 (1H, m), 3.32 and 3.72 (2H, 2 × d, *J* = 13.8 Hz), 3.96 and 4.79 (2H, 2 × d × d, *J* = 14.3, 8.2, 3.3 Hz), 6.97–7.45 (7H, m), 7.66 (1H, s), 7.70–7.74 (2H, m). ^{13}C NMR (75 MHz, $CDCl_3$): δ 32.9, 38.2, 53.4, 61.3, 119.9, 125.8, 127.1, 128.1, 128.8, 129.0, 129.5, 130.2, 130.8, 133.5, 135.9, 147.9. IR (cm^{-1}): ν_{max} = 2922, 1464, 1441, 1358, 1051, 768, 751, 753, 692. MS (70 eV) *m/z* (%): 325/7 (M^+ + 1, 100). Anal. calcd for $C_{18}H_{17}ClN_4$: C 66.56, H 5.28, N 17.25; found: C 66.35, H 5.65, N 17.16. Mp = 102.7 °C.

Synthesis of 2,3-diamino-1-(1,2,3-triazol-1-yl)propanes 16

General procedure. In a 10 mL microwave recipient, 1-arylmethyl-2-[(1,2,3-triazol-1-yl)methyl]aziridine (**15**) (0.344 mmol), diethylamine hydrochloride (6.88 mmol) and diethylamine (3.44 mmol) were dissolved in acetonitrile (6 mL). The resulting mixture was put in the microwave for 2 h at 140 °C. Afterwards, the reaction mixture was neutralized using a saturated sodium bicarbonate solution (10 mL), poured into water (15 mL) and extracted with Et_2O (3 × 10 mL). The combined organic fractions were washed with brine (3 × 10 mL). Drying (K_2CO_3), filtration of the drying agent and removal of the solvent in vacuo afforded 2,3-diamino-1-(1,2,3-triazol-1-yl)propane (**16**), which was purified by means of column chromatography on silica gel.

2-[(4-Chlorophenyl)methyl]amino-3-diethylamino-1-(4-phenyl-1,2,3-triazol-1-yl)propane (16b)

R_f = 0.30 ($CHCl_3$/MeOH 95/5), light-brown oil, 60%. ^{1}H NMR (300 MHz, $CDCl_3$): δ 0.94 (6H, t, *J* = 7.2 Hz), 2.30–2.53 (6H, m), 3.04–3.11 (1H, m), 3.68 and 3.76 (2H, 2 × d, *J* = 13.2 Hz), 4.36 and 4.46 (2H, 2 × d × d, *J* = 14.0, 5.2, 4.7 Hz), 7.19–7.46 and 7.81–7.84 (9H, m), 7.84 (1H, s). ^{13}C NMR (75 MHz, $CDCl_3$): δ 11.8, 47.1, 51.2, 52.4, 55.0, 55.3, 121.1, 125.8, 128.2, 128.7, 129.0, 129.5, 130.8, 132.9, 138.7, 147.6. IR (cm^{-1}): ν_{max} = 2968, 1490, 1464, 1088, 1074, 760, 694. MS (70 eV) *m/z* (%): 398/400 (M^+ + 1, 100). HRMS (ESI) calcd for $C_{22}H_{29}ClN_5$ 398.2111 $[M + H]^+$, found 398.2105.

3-Diethylamino-1-(4-phenyl-1,2,3-triazol-1-yl)-2-[(4-methoxyphenyl)methyl]amino-propane (16c)

R_f = 0.21 ($CHCl_3$/MeOH 96/4), light-brown oil, 56%. ^{1}H NMR (300 MHz, $CDCl_3$): δ 0.93 (6H, t, *J* = 7.2 Hz), 2.25–2.52 (6H, m), 3.04–3.12 (1H, m), 3.68 and 3.75 (2H, 2 × d, *J* =

13.2 Hz), 3.76 (3H, s); 4.36 and 4.44 (2H, 2 × d × d, J = 13.9, 5.3, 4.4 Hz), 6.82–6.86 (2H, m), 7.18–7.45 (5H, m), 7.83–7.85 (2H, m), 7.86 (1H, s). ^{13}C NMR (75 MHz, $CDCl_3$): δ 11.8, 47.0, 51.4, 52.3, 54.9, 55.3, 114.0, 121.2, 125.8, 128.1, 128.9, 129.4, 130.9, 132.3, 147.5, 158.8. IR (cm^{-1}): ν_{max} = 2967, 1511, 1246, 1034, 762, 694. MS (70 eV) m/z (%): 394 (M^+ + 1, 100). HRMS (ESI) calcd for $C_{23}H_{32}N_5O$ 394.2607 $[M + H]^+$, found 394.2602.

3-Diethylamino-2-(phenylmethyl)amino-1-[4-(4-methylphenyl)-1,2,3-triazol-1-yl]propane (16d)

R_f = 0.21 ($CHCl_3$/MeOH 96/4), light-brown oil, 53%. ^{1}H NMR (300 MHz, $CDCl_3$): δ 0.91 (6H, t, J = 7.2 Hz), 2.25–2.51 (6H, m), 2.36 (3H, s), 3.04–3.12 (1H, m), 3.70 and 3.80 (2H, 2 × d, J = 13.5 Hz), 4.34 and 4.42 (2H, 2 × d × d, J = 14.0, 5.5, 4.4 Hz), 7.20–7.33 (7H, m), 7.71–7.73 (2H, m), 7.83 (1H, s). ^{13}C NMR (75 MHz, $CDCl_3$): δ 11.8, 21.4, 47.1, 51.9, 52.2, 54.9, 55.3, 120.9, 125.7, 127.2 128.3, 128.6, 129.6, 128.1, 137.9, 140.2, 147.5. IR (cm^{-1}): ν_{max} = 2925, 1454, 751, 698. MS (70 eV) m/z (%): 378 (M^+ + 1, 100). HRMS (ESI) calcd for $C_{23}H_{32}N_5$ 378.2658 $[M + H]^+$, found 378.2646.

3-Diethylamino-2-(phenylmethyl)amino-1-[4-(4-methoxyphenyl)-1,2,3-triazol-1-yl]propane (16e)

R_f = 0.23 ($CHCl_3$/MeOH 96/4), light-brown oil, 85%. ^{1}H NMR (300 MHz, $CDCl_3$): δ 0.93 (6H, t, J = 7.2 Hz), 2.26–2.52 (6H, m), 3.05–3.13 (1H, m), 3.73 and 3.82 (2H, 2 × d, J = 14.0 Hz), 3.85 (3H, s), 4.37 and 4.44 (2H, 2 × d × d, J = 13.8, 5.0, 4.4 Hz), 6.94–6.99 (2H, m), 7.21–7.35 (5H, m), 7.73–7.78 (2H, m), 7.78 (1H, s). ^{13}C NMR (75 MHz, $CDCl_3$): δ 11.8, 47.0, 52.0, 52.3 55.0, 55.4, 55.4, 114.3, 120.3, 123.6, 127.1, 127.2, 128.2, 128.6, 140.2, 147.4, 159.6. IR (cm^{-1}): ν_{max} = 2968, 1498, 1455, 1247, 1175, 1030, 835, 750, 698. MS (70 eV) m/z (%): 394 (M^+ + 1, 100). HRMS (ESI) calcd for $C_{23}H_{32}N_5O$ 394.2607 $[M + H]^+$, found 394.2599.

2-[(2-Chlorophenyl)methyl]amino-3-dimethylamino-1-(4-phenyl-1,2,3-triazol-1-yl)propane (16f)

R_f = 0.22 ($CHCl_3$/MeOH 95/5), light-brown oil, 57%. ^{1}H NMR (300 MHz, $CDCl_3$): δ 2.05–2.23 (2H, m), 2.10 (6H, s), 3.07–3.14 (1H, m), 3.86 and 3.96 (2H, 2 × d, J = 13.8 Hz), 4.44 and 4.50 (2H, 2 × d × d, J = 14.2, 4.7, 4.4 Hz), 7.18–7.45 (7H, m), 7.81–7.85 (2H, m), 7.93 (1H, s). ^{13}C NMR (75 MHz, $CDCl_3$): δ 45.6, 49.3, 51.9, 54.5, 61.0, 121.3, 125.8, 127.1, 128.1, 128.7, 128.9, 129.7, 130.5, 130.9, 134.0, 137.4, 147.6. IR (cm^{-1}): ν_{max} = 2943, 2822, 1462, 1442, 750, 694. MS (70 eV) m/z (%): 370/2 (M^+ + 1, 100). HRMS (ESI) calcd for $C_{20}H_{25}ClN_5$ 370.1798 $[M + H]^+$, found 370.1789.

3-Dimethylamino-2-(phenylmethyl)amino-1-(4-phenyl-1,2,3-triazol-1-yl)propane (16g)

R_f = 0.24 ($CHCl_3$/MeOH 95/5), light-brown oil, 60%. ^1H NMR (300 MHz, $CDCl_3$): δ 2.05–2.26 (2H, m), 2.14 (6H, s), 3.08–3.16 (1H, m), 3.77 and 3.89 (2H, 2 × d, *J* = 13.5 Hz), 4.41 and 4.48 (2H, 2 × d × d, *J* = 14.4, 5.0, 4.7 Hz), 7.21–7.46 (8H, m), 7.82–7.86 (2H, m), 7.87 (1H, s). ^{13}C NMR (75 MHz, $CDCl_3$): δ 45.7, 51.7, 51.9, 54.6, 61.1, 121.2, 125.8, 127.3, 128.1, 128.3, 128.6, 128.9, 130.9, 140.1, 147.5. IR (cm^{-1}): ν_{max} = 2943, 2822, 1460, 1044, 1028, 761, 751, 694. MS (70 eV) *m*/*z* (%): 336 (M^+ + 1, 100). HRMS (ESI) calcd for $C_{20}H_{26}N_5$ 336.2188 $[M + H]^+$, found 336.2187.

References

1. D’hooghe, M.; Mollet, K.; Dekeukeleire, S.; De Kimpe, N. *Org. Biomol. Chem.* **2010,** *8,* 607–615. doi:10.1039/b919864d

2. De Kimpe, N.; Jolie, R.; De Smaele, D. *J. Chem. Soc. Chem. Commun.* **1994,** 1221–1222. doi:10.1039/c39940001221

3. D’hooghe, M.; Waterinckx, A.; Kimpe, N. *J. Org. Chem.* **2005,** *70,* 227–232. doi:10.1021/jo048486f

4. D’hooghe, M.; Rottiers, M.; Jolie, R.; De Kimpe, N. *Synlett* **2005,** 931–934. doi:10.1055/s-2005-864801
